# Development of an early auxiliary diagnostic model for disseminated tuberculosis in HIV-negative tuberculosis patients

**DOI:** 10.3389/fpubh.2026.1779279

**Published:** 2026-08-05

**Authors:** Yicheng Guo, Xingyu Liu, Chen Yang, Shuanglin Gu, Weiwei Gao, Yi Zeng

**Affiliations:** 1 Department of Tuberculosis, The School of Public Health of Nanjing Medical University, The Second Hospital of Nanjing, Nanjing, China; 2 Department of Tuberculosis, The Second Hospital of Nanjing, Nanjing, China; 3 Department of Clinical Research Center, The Second Hospital of Nanjing, Nanjing University of Chinese Medicine, Nanjing, China

**Keywords:** diagnostic algorithm, disseminated tuberculosis, immune indicators, machine learning, model development

## Abstract

**Background:**

Disseminated tuberculosis (DI-TB) can induce multiple organ dysfunction syndrome and carries a high mortality rate. This study aimed to establish an early-warning diagnostic model to facilitate the identification of DI-TB high-risk patients and improve clinical prognosis.

**Methods:**

We retrospectively enrolled 1,122 HIV-negative hospitalized patients with disseminated tuberculosis (DI-TB) or pulmonary tuberculosis (PTB) admitted to Nanjing Second Hospital. Using conventional laboratory tests and multidimensional lymphocyte subset analysis, a diagnostic model was constructed employing machine learning algorithms. Feature importance analysis was performed using the Shapley Additive Explanations (SHAP) framework.

**Results:**

We screened nine significant features for model development. The XGB model demonstrated favorable diagnostic performance in the internal test set, achieving an AUC of 0.983, a Brier score of 0.025, an accuracy of 0.949, sensitivity of 0.914, specificity of 0.961, F1-score of 0.897, MCC of 0.864, NPV of 0.972, and PPV of 0.881. The SHAP interpretability system ranked features by importance, illustrating their positive or negative contributions to XGB predictions and explaining how individual features influenced model outputs.

**Conclusion:**

This study developed and internally evaluated a machine learning-enabled auxiliary risk-stratification model for identifying dissemination risk among HIV-negative hospitalized TB patients. The XGB model showed favorable internal performance and may provide a potential auxiliary tool for early DI-TB risk assessment, but prospective external validation is still required before routine clinical application.

## 1 Introduction

Tuberculosis (TB) is a chronic infectious disease caused by *Mycobacterium tuberculosis* (MTB) infection. According to the 2024 WHO Global Tuberculosis Report (1), the global TB incidence in 2023 was 134 per 100,000 population, representing a marginal increase of 0.2% compared with 2022. TB-related deaths numbered 1.25 million in 2023, continuing the downward trend observed since 2022. From 2015 to 2023, global TB incidence declined by only 8.3%, falling far short of the WHO End TB Strategy milestone of a 50% reduction by 2025 and indicating a substantial gap toward the goal of TB elimination. The persistently high incidence and prevalence signify a substantial burden of TB cases, among which a subset presents with severe disease manifestations and disseminated lesions. These complex cases complicate clinical management, posing significant challenges to TB prevention and control efforts.

DI-TB is defined by the presence of confirmed TB lesions in two or more non-contiguous organs (2). DI-TB typically originates from hematogenous or lymphatic spread of MTB, arising either from primary pulmonary foci or through reactivation and dissemination of latent foci (3). As the most severe form of TB—particularly when hematogenous dissemination involves concurrent neurological, skeletal, or gastrointestinal involvement—DI-TB can lead to life-threatening organ dysfunction or death if not promptly diagnosed and treated. Studies demonstrate significantly higher mortality in DI-TB compared to localized pulmonary TB (PTB) (4). However, the nonspecific clinical manifestations of DI-TB often delay diagnosis and treatment, resulting in poor patient prognoses (5). Thus, early diagnosis and identification of high-risk individuals are critical.

Conventional laboratory parameters and lymphocyte profiling data possess underutilized diagnostic potential. Previous studies (6–8) have confirmed the auxiliary role of such biomarkers in TB differential diagnosis. Although logistic regression models have preliminarily revealed correlations between T-lymphocyte subsets and DI-TB progression (9), a multidimensional analysis integrating B-lymphocyte phenotypes, cytokine profiles, and disease-activity markers remains lacking. A systematic evaluation of these immune indicators would not only holistically characterize host immune responses but also provide quantitative evidence for dynamic disease monitoring.

As a core artificial intelligence technology, machine learning (ML) excels at identifying latent patterns within high-dimensional datasets, offering potential advantages in clinical prediction tasks. This study integrates routine laboratory parameters with lymphocyte immunophenotypic profiles to develop an ML-based auxiliary risk-stratification model for DI-TB. Clinically, this model may help identify HIV-negative hospitalized TB patients at higher risk of dissemination and support clinicians in selecting patients who may benefit from further multi-organ evaluation. However, its clinical utility requires prospective external validation before routine implementation.

## 2 Method

### 2.1 Study population

**Figure 1:**
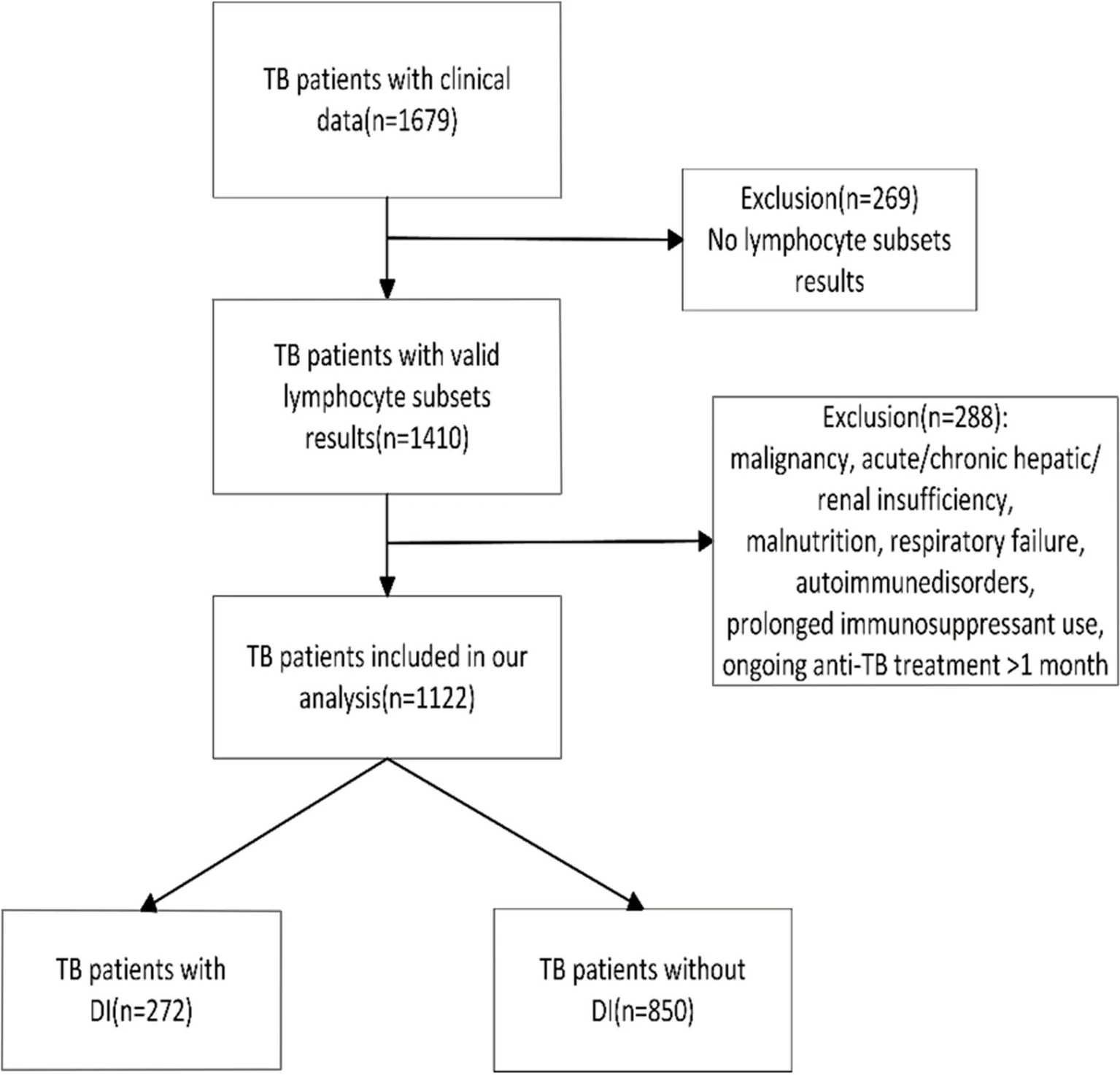
Flowchart of the study population.

This retrospective study analyzed the clinical records of HIV-negative patients with tuberculosis admitted to Nanjing Second Hospital between January 2023 and June 2025. From an initial cohort of 1,679 HIV-negative TB inpatients, 557 patients were excluded based on predefined criteria: incomplete lymphocyte subset testing, major comorbidities (malignancy, acute/chronic hepatic/renal insufficiency, malnutrition, respiratory failure, autoimmune disorders), prolonged immunosuppressant use, or ongoing anti-TB treatment >1 month. The final analysis included 1,122 eligible patients. The subject screening process is illustrated in Figure 1. Lymphocyte subset testing was not routinely performed for all admitted TB patients during the study period. Because lymphocyte subset parameters were key candidate predictors in this study, patients without these measurements were excluded from model development.

This was an observational retrospective study using anonymized clinical data. The requirement for written informed consent was waived by the Human Research Ethics and System Review Committee of the Second Hospital of Nanjing because of the retrospective nature of the study. The study received approval from the Human Research Ethics and System Review Committee of the Second Hospital of Nanjing (ID: 2025-LS-ky034).

### 2.2 Case definitions

PTB diagnosis required ≥1 of the following: Positive sputum/bronchoalveolar lavage fluid (BALF) culture; Acid-fast bacilli (AFB) smear positivity in sputum/BALF with corroborative radiographic evidence; Positive GeneXpert MTB/RIF assay (sputum/BALF); Histopathological confirmation of PTB; Typical TB imaging or immunological findings (e.g., T-SPOT. TB); Clinical response consistent with PTB following anti-TB therapy.

DI-TB was defined as tuberculosis involving two or more non-contiguous organs or anatomical sites. The diagnosis was determined by two experienced tuberculosis physicians based on microbiological, molecular, pathological, radiological, and clinical evidence. Microbiological confirmation was not required for every extrapulmonary site, because obtaining specimens from all involved organs was not always clinically feasible. Evidence for extrapulmonary involvement included positive mycobacterial culture, positive acid-fast bacilli smear, positive GeneXpert MTB/RIF assay, histopathological findings compatible with tuberculosis, or typical imaging findings combined with clinical manifestations, immunological evidence, and response to anti-tuberculosis treatment. Imaging-only evidence was accepted only when the imaging findings were highly compatible with tuberculosis and were supported by clinical and laboratory evidence after multidisciplinary review. Multifocal pulmonary lesions confined to the lungs were classified as PTB rather than DI-TB unless there was evidence of hematogenous or lymphatic dissemination beyond the pulmonary system. The same operational diagnostic criteria were applied uniformly to all patients during outcome classification.

### 2.3 Data collection

The Central Laboratory of Nanjing Second Hospital conducted pathogenetic assays (smear microscopy, culture, drug susceptibility testing, and GeneXpert MTB/RIF), molecular assays, and immunological assays (interferon-gamma release assays). Lymphocyte subset and cytokine indicators included CD3 + total T cells%, CD3 + total T cells, CD8+/CD45+, CD8 + T cells, CD4+/CD45+, CD4 + T cells, CD4 + CD8+/CD45+, CD4 + CD8 + T cells, CD16/56 + NK%, CD16/56 + NK cells, CD19 + B cells, CD45 + lymphocyte count, Th/Ts (CD4+/CD8+), CD19 + B%, IL-2, IL-4, IL-6, IL-10, IFN-*γ*, TNF-*α*, IL-17A, IL-1β, IFN-α, IL-5, IL-12p70, and IL-8. Complete blood count, biochemical, coagulation, and inflammatory markers included high-sensitivity C-reactive protein (hs-CRP), basophil count (BASO), basophil percentage (BASO%), eosinophil count (EOS), eosinophil percentage (EOS%), hematocrit (HCT), hemoglobin (Hb), lymphocyte count (Lymph), lymphocyte percentage (Lymph%), mean corpuscular hemoglobin (MCH), mean corpuscular hemoglobin concentration (MCHC), mean corpuscular volume (MCV), monocyte count (Mono), monocyte percentage (Mono%), mean platelet volume (MPV), neutrophil count (ANC), neutrophil percentage (GR%), plateletcrit (PCT), platelet distribution width (PDW), platelet count (PLT), red blood cell count (RBC), red blood cell distribution width (RDW-CV), and total white blood cell count (WBC). The above indicators were obtained from the hospital information system.

### 2.4 Overview of machine learning models

Logistic Regression (LR), a generalized linear model, maps features to binary/multiclass probabilities via the Sigmoid function. Its strengths lie in parameter interpretability (quantified as odds ratios, OR) and applicability to small-sample scenarios, though it cannot capture nonlinear interactions. Random Forest (RF) employs a bootstrap aggregation (bagging) ensemble strategy, constructing multiple decision trees for collective voting. This approach confers robustness against noise while enabling feature importance evaluation. Support Vector Machine (SVM) operates on the structural risk minimization principle, leveraging kernel functions (e.g., Radial Basis Function, RBF) to project low-dimensional nonlinear problems into high-dimensional spaces for linear separation. SVM demonstrates exceptional generalization performance in small-sample contexts. XGBoost (XGB) iteratively optimizes Classification and Regression Trees (CART) through a gradient boosting framework. By incorporating second-order derivative approximations of the loss function with regularization techniques (e.g., L1/L2 penalties), it excels in tabular data prediction tasks but exhibits sensitivity to hyperparameters and requires training time–cost tradeoffs. K-Nearest Neighbors (KNN), a non-parametric lazy learning algorithm, performs local voting classification based on distance metrics (e.g., Euclidean distance, cosine similarity), yielding flexible decision boundaries. Each of these algorithms possesses distinct advantages for feature optimization. To comparatively evaluate machine learning method performance, we implemented five models: LR, RF, SVM, XGB, and KNN (10–14).

### 2.5 Statistical analysis

Continuous variables are expressed as medians with interquartile ranges (IQR). Comparisons between continuous variables employed the Student’s *t*-test assuming normality and homogeneity of variance, or the Mann–Whitney *U* test otherwise. Categorical variables are presented as numbers (percentages) and compared using the χ^2^ test or Fisher’s exact test. Two-tailed *p* values <0.05 were considered statistically significant.

To minimize potential information leakage, all model-development procedures were performed after stratified data splitting. The full dataset was first divided into a training set and an internal test set at a ratio of 7:3. Feature screening, SMOTE oversampling, LASSO regression, backward stepwise multivariable logistic regression, hyperparameter tuning, and model fitting were conducted only within the training set. The internal test set was not used for feature selection, parameter tuning, or model optimization, and was used only for final performance evaluation. Patients with missing lymphocyte subset testing were excluded according to the predefined exclusion criteria. For the remaining variables included in model development, complete-case analysis was performed. To address class imbalance between DI-TB and PTB cases, SMOTE with k = 5 nearest neighbors was used as the predefined resampling strategy during model development. To evaluate whether the use of SMOTE affected model robustness, a sensitivity comparison was performed between the XGB model trained with the predefined SMOTE k = 5 setting and the XGB model trained without SMOTE. The initial candidate predictor pool included demographic characteristics, clinical symptoms, comorbidities, routine hematological indicators, biochemical and inflammatory markers, cytokines, and lymphocyte subset parameters. To identify independent risk factors for DI-TB, backward stepwise regression was first used for variable reduction, and the retained variables were then entered into a multivariable logistic regression model. Variables that remained statistically significant in the multivariable logistic regression model were considered independent risk factors for DI-TB. For ML model construction, LASSO regression was further applied to the training set for dimensionality reduction under the λ1se criterion. The final modeling variables were defined as the overlapping variables between the predictors retained by backward stepwise regression and those selected by LASSO regression. Hyperparameter tuning was conducted using grid search with five-fold cross-validation, with cross-validated AUC as the optimization criterion. Model performance was evaluated using AUC, sensitivity, specificity, accuracy, PPV, NPV, F1-score, MCC, and Brier score. A confusion matrix was generated for the final XGB model in the internal test set to further describe its classification performance. The matrix was based on the default predicted class labels generated by the XGB model rather than a manually specified clinical probability threshold. Calibration plots and Brier scores were used to assess the agreement between predicted probabilities and observed outcomes. DCA was performed to evaluate the net clinical benefit across different threshold probabilities. SHAP analysis was used to interpret feature-specific contributions to model predictions. Reporting of this prediction model study was guided by relevant TRIPOD+AI principles where applicable. A completed TRIPOD+AI checklist is provided in Supplementary Table S5. PROBAST-related risk-of-bias domains, including participant selection, predictor assessment, outcome definition, sample size, missing data, model evaluation, and overfitting, were considered when interpreting the findings. All analyses were performed using R software (version 4.4.1).

## 3 Results

### 3.1 Participant characteristics

The baseline characteristics are presented in Table 1. The study enrolled 1,122 HIV-negative hospitalized tuberculosis patients, including 850 PTB cases (75.8%) and 272 DI-TB cases (24.2%). Key findings revealed: DI-TB patients were significantly older than PTB patients (median age: 60.0 vs. 46.0 years; *p* < 0.001) with higher male predominance (65.4% vs. 55.4%; *p* = 0.004). The DI-TB group exhibited greater weight loss (21.3% vs. 8.6%; *p* < 0.001) and fever incidence (38.6% vs. 23.5%; p < 0.001) while cough showed no intergroup difference (67.3% vs. 68.0%; *p* = 0.883). Patients with DI-TB had higher overall comorbidities (40.1% vs. 25.1%; p < 0.001), particularly coronary heart disease (5.5% vs. 2.4%; *p* = 0.016). Hypertension demonstrated borderline significance (21.3% vs. 16.1%; *p* = 0.060), and diabetes lacked statistical difference (14.3% vs. 15.1%; *p* = 0.847). Smoking (overall 13.4%) and alcohol use (overall 6.6%) showed no significant intergroup variation (*p* > 0.05). These findings characterize patients with DI-TB as predominantly older males with prominent systemic wasting symptoms and cardiovascular comorbidity burden.

**Table 1:** Baseline characteristics.

Variable	Level	Overall	PTB	DI-TB	*P* value
*n*		1,122	850	272	
Sex (%)	F	473 (42.16)	379 (44.59)	94 (34.56)	0.004
	M	649 (57.84)	471 (55.41)	178 (65.44)	
Age (median [IQR])		51.000 [31.000, 65.000]	46.000 [29.000, 61.000]	60.000 [43.000, 73.000]	<0.001
Smoking (%)	No	972 (86.63)	741 (87.18)	231 (84.93)	0.397
	Yes	150 (13.37)	109 (12.82)	41 (15.07)	
Drinking (%)	No	1,048 (93.40)	795 (93.53)	253 (93.01)	0.875
	Yes	74 (6.60)	55 (6.47)	19 (6.99)	
Fever (%)	No	817 (72.82)	650 (76.47)	167 (61.40)	<0.001
	Yes	305 (27.18)	200 (23.53)	105 (38.60)	
Cough (%)	No	361 (32.17)	272 (32.00)	89 (32.72)	0.883
	Yes	761 (67.83)	578 (68.00)	183 (67.28)	
Weight loss (%)	No	991 (88.32)	777 (91.41)	214 (78.68)	<0.001
	Yes	131 (11.68)	73 (8.59)	58 (21.32)	
Underlying disease (%)	No	800 (71.30)	637 (74.94)	163 (59.93)	<0.001
	Yes	322 (28.70)	213 (25.06)	109 (40.07)	
Hypertension (%)	No	927 (82.62)	713 (83.88)	214 (78.68)	0.060
	Yes	195 (17.38)	137 (16.12)	58 (21.32)	
Diabetes (%)	No	955 (85.12)	722 (84.94)	233 (85.66)	0.847
	Yes	167 (14.88)	128 (15.06)	39 (14.34)	
Coronary heart disease (%)	No	1,087 (96.88)	830 (97.65)	257 (94.49)	0.016
	Yes	35 (3.12)	20 (2.35)	15 (5.51)	

### 3.2 Independent risk factors

Furthermore, we explored the independent risk factors for DI-TB. Backward stepwise regression was first used for variable reduction, and 21 retained variables were then entered into a multivariable logistic regression model. This analysis identified 14 independent risk factors significantly associated with DI-TB, including sex, smoking, drinking, Lymph, Mono%, ANC, WBC, IL-2, IFN-*γ*, CD3 + total T cells, CD8+/CD45+, CD8 + T cells, CD4 + T cells, and CD19 + B% (Supplementary Table S1).

### 3.3 Feature selection

For ML model construction, LASSO regression was applied to the training set to further reduce dimensionality and improve model parsimony. Regularization penalties were used to reduce overfitting and severe multicollinearity. The optimal penalty parameter *λ* was determined through cross-validation, and the λ1se criterion was selected to enforce stronger regularization and improve model stability. Under the λ1se criterion, LASSO regression selected a subset of predictors with non-zero coefficients. The final modeling variables were obtained by taking the intersection between the LASSO-selected predictors and the variables retained by backward stepwise regression. This intersection-based strategy yielded nine variables for subsequent model development (Supplementary Figure S1).

### 3.4 Establishment and evaluation of diagnostic models

Predictive models, including LR, KNN, SVM, RF, and XGB, were constructed using the selected features. During model development, grid search combined with five-fold cross-validation was applied for hyperparameter optimization. This approach was used to reduce overfitting and evaluate model stability within the internal cohort; however, it could not establish external generalizability.

**Figure 2:**
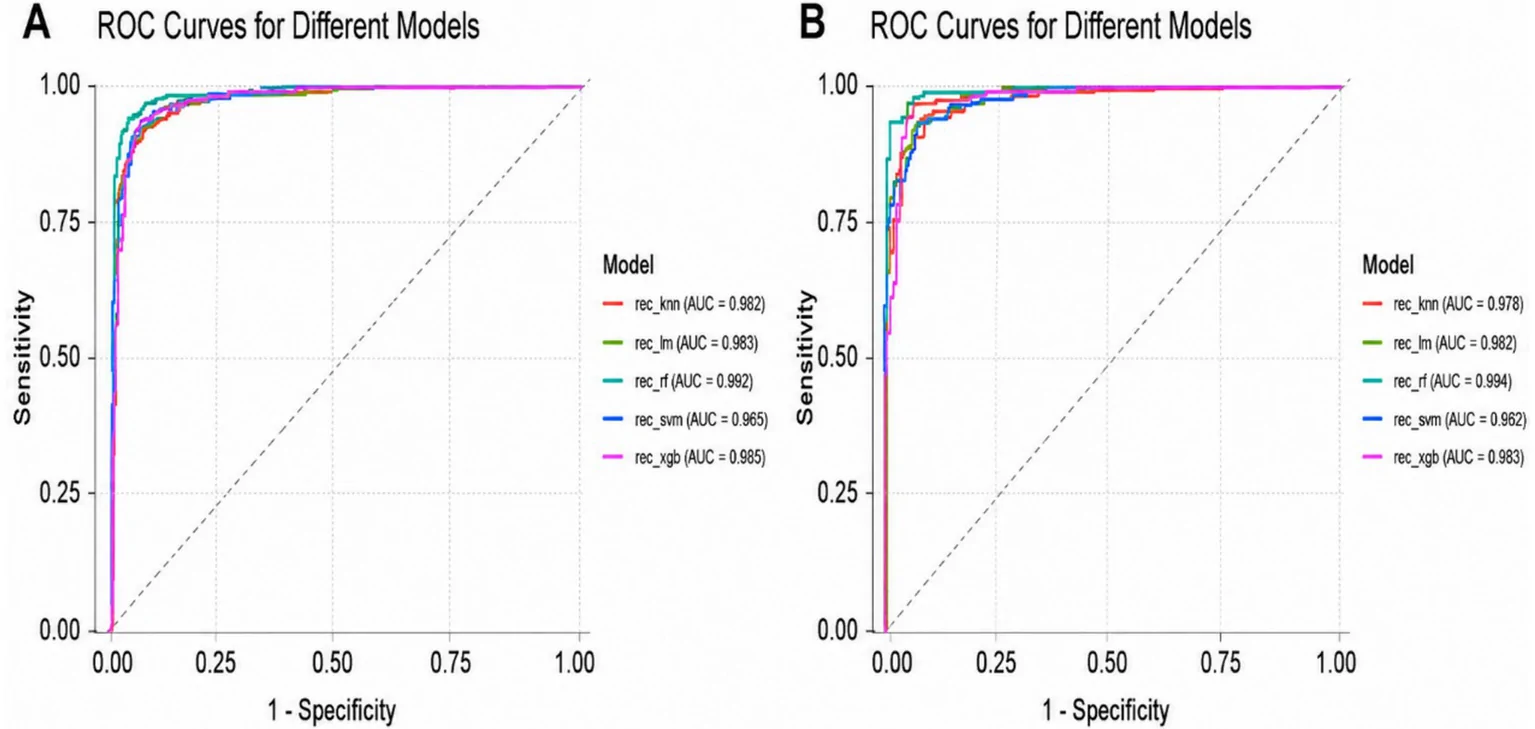
Receiver operating characteristic (ROC) curves of five ML models in the training and internal test sets **(A)** Training set; **(B)** Internal test set. LR, logistic regression; KNN, *k*-nearest neighbors; SVM, support vector machine; RF, random forest; XGB, extreme gradient boosting.

As shown in Figure 2, all five models demonstrated good discriminative performance in both the training and internal test sets. In the training set, XGB achieved the highest AUC of 0.991, followed by RF (AUC = 0.989), LR (AUC = 0.988), SVM (AUC = 0.984), and KNN (AUC = 0.970). In the internal test set, XGB also showed the highest AUC of 0.983, followed by RF and LR (both AUC = 0.981), SVM (AUC = 0.977), and KNN (AUC = 0.955).

As shown in Supplementary Figure S2, calibration analysis was performed to evaluate the agreement between predicted probabilities and observed DI-TB proportions. In both the training and internal test sets, the calibration curves of the five models were generally close to the 45-degree reference line, indicating acceptable agreement between predicted and observed risks. In the internal test set, XGB showed the lowest Brier score of 0.025, followed closely by RF with a Brier score of 0.026. These findings suggest that XGB provided the most favorable discrimination and probabilistic prediction performance among the five models.

The classification metrics of the five models are summarized in Supplementary Table S2. In the internal test set, XGB achieved an accuracy of 0.949, sensitivity of 0.914, specificity of 0.961, PPV of 0.881, NPV of 0.972, F1-score of 0.897, and MCC of 0.864. The confusion matrix of the final XGB model is presented in Supplementary Table S3. Among 255 PTB patients in the internal test set, 245 were correctly classified as PTB and 10 were misclassified as DI-TB. Among 81 DI-TB patients, 74 were correctly classified as DI-TB and 7 were misclassified as PTB.

To further evaluate clinical utility, DCA was performed on the internal test set. As shown in Supplementary Figure S3, all five ML models achieved higher net benefit than the “treat-none” strategy across most threshold probabilities. XGB demonstrated relatively higher net benefits across a broad clinically relevant threshold range. Overall, considering discrimination, calibration, DCA, and classification performance, XGB was selected as the final model.

### 3.5 Sensitivity comparison with and without SMOTE

To further evaluate whether the use of SMOTE influenced XGB model robustness, a sensitivity comparison was performed between the XGB model trained without SMOTE and the XGB model trained with the predefined SMOTE k = 5 setting. SMOTE was applied only to the training set after stratified data splitting, while the internal test set retained its original class distribution. As shown in Supplementary Table S4, the XGB model trained without SMOTE achieved an AUC of 0.975, accuracy of 0.961, sensitivity of 0.889, specificity of 0.984, PPV of 0.947, NPV of 0.965, F1-score of 0.917, MCC of 0.893, and Brier score of 0.034. After applying SMOTE, the XGB model showed a slightly higher AUC (0.983) and sensitivity (0.914), with an NPV of 0.972, while specificity, PPV, F1-score, MCC, and Brier score showed small decreases. These findings suggest that SMOTE did not artificially inflate overall model performance but slightly improved sensitivity for identifying DI-TB cases.

### 3.6 Interpretability analysis

**Figure 3:**
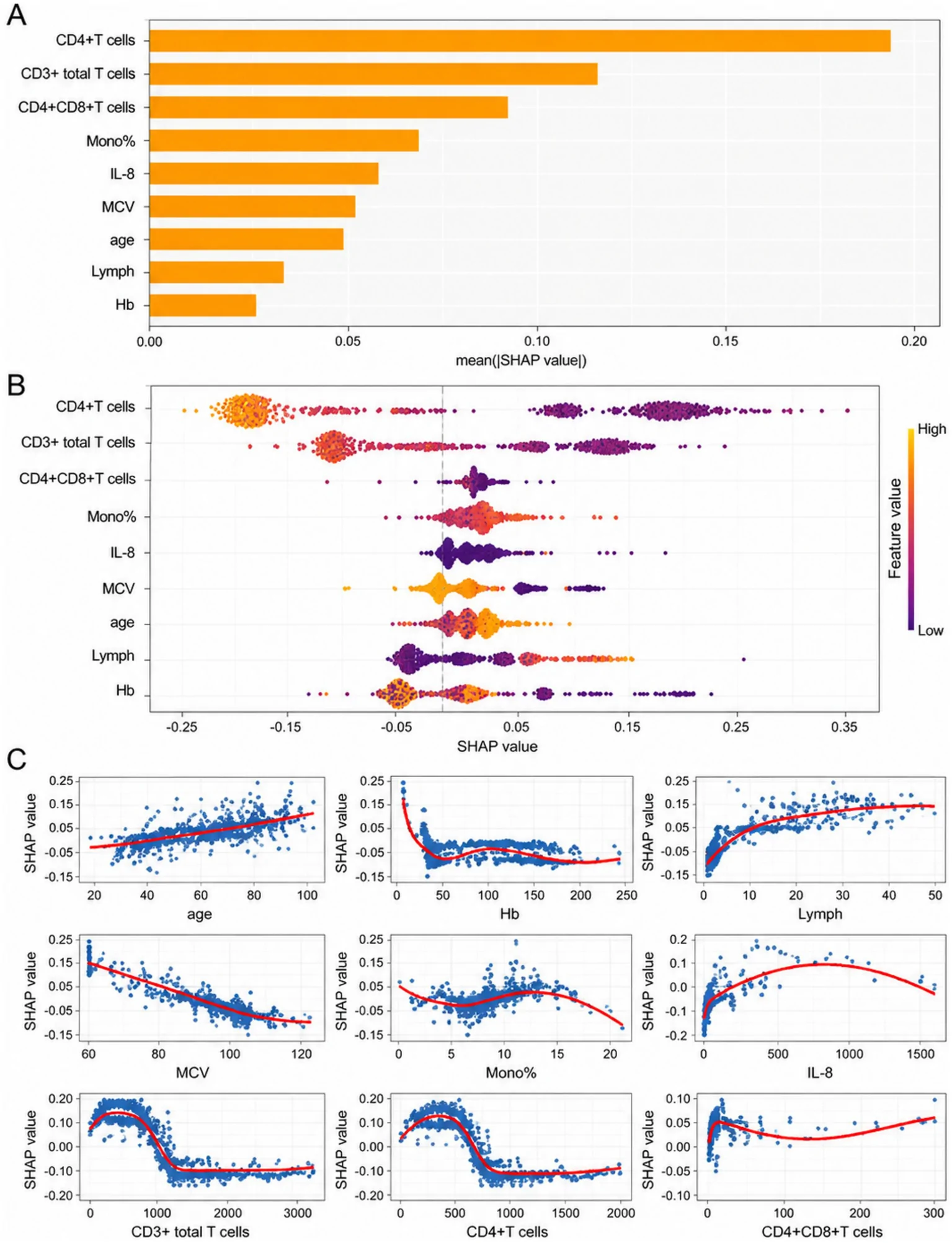
SHAP-based interpretability analysis of the final XGB model. **(A)** Mean absolute SHAP values of the nine selected predictors. **(B)** SHAP beeswarm plot showing the direction and distribution of feature effects. **(C)** SHAP dependence plots showing the relationship between individual predictors and SHAP values.

To explore the impact and functional patterns of clinical features on XGB model predictions, this study employed SHAP (Shapley Additive Explanations) analysis, with results visualized in Figure 3. The feature importance plot (Figure 3A) displays the mean absolute SHAP values, revealing CD4 + T cells as the most influential factor, followed by CD3 + total T cells, CD4 + CD8 + T cells, and other variables, while Hb showed the weakest effect. This indicates that immune cell-related indicators played an important role in XGB model decisions. Subsequently, the SHAP beeswarm plot (Figure 3B) illustrates the distribution of individual SHAP values across feature magnitudes. Higher SHAP values denote greater feature impact on the XGB-based diagnostic model. Notably, advanced age, elevated Lymph, IL-8, Hb, MCV, Mono%, CD3 + total T cells, CD4 + T cells, and CD4 + CD8 + T cells were associated with increased predicted probabilities of DI-TB. Furthermore, SHAP dependence plots (Figure 3C) delineated the relationship between individual features and SHAP values. Collectively, these visualizations provided multidimensional evidence for interpreting the contribution of immune, hematological, and inflammatory features to XGB model predictions.

### 3.7 Subgroup analysis of model performance

To further assess the internal stability of the final XGB model across clinically relevant subgroups, subgroup analyses were performed in the internal test set according to age, sex, and diabetes status. As shown in Table 2, the XGB model maintained favorable discriminative performance across all predefined subgroups.

**Table 2:** Subgroup performance of the XGB model in the internal test set.

Subgroup variable	Subgroup level	Internal test set AUC (95% CI)
Age	<40 years	0.995 (0.987–1.000)
Age	40–60 years	0.970 (0.937–0.999)
Age	≥60 years	0.973 (0.934–1.000)
Sex	Female	0.946 (0.871–1.000)
Sex	Male	0.991 (0.979–0.999)
Diabetes	No	0.982 (0.959–0.999)
Diabetes	Yes	0.980 (0.940–1.000)

AUC, area under the receiver operating characteristic curve; CI, confidence interval; XGB, extreme gradient boosting.

In the age subgroups, the AUCs were 0.995 (95% CI: 0.987–1.000) for patients aged <40 years, 0.970 (95% CI: 0.937–0.999) for patients aged 40–60 years, and 0.973 (95% CI: 0.934–1.000) for patients aged ≥60 years. In the sex subgroups, the AUC was 0.946 (95% CI: 0.871–1.000) in female patients and 0.991 (95% CI: 0.979–0.999) in male patients. For diabetes status, the AUCs were 0.982 (95% CI: 0.959–0.999) in patients without diabetes and 0.980 (95% CI: 0.940–1.000) in patients with diabetes.

Overall, the XGB model showed favorable discriminative performance across age, sex, and diabetes subgroups in the internal test set. The AUC was numerically lower in female patients and in patients aged 40–60 or ≥60 years than in the corresponding comparison subgroups, but the confidence intervals were relatively wide and largely overlapping. Therefore, these subgroup results should be interpreted as exploratory. Larger external validation cohorts are needed to further evaluate the stability and generalizability of the XGB model across different clinical populations.

## 4 Discussion

This retrospective study included 1,122 hospitalized tuberculosis patients, among whom 272 had DI-TB. Analysis of clinical parameters, laboratory biochemical markers, and immunological indicators revealed 14 independent risk factors for DI-TB through multivariable logistic regression, including sex, smoking, drinking, CD3 + total T cells, CD8+/CD45+, CD8 + T cells, CD4 + T cells, CD19 + B%, and others. Subsequently, five ML models were constructed. In the internal test set, XGB achieved the highest AUC (0.983) and the lowest Brier score (0.025), with an accuracy of 0.949, sensitivity of 0.914, specificity of 0.961, F1-score of 0.897, MCC of 0.864, NPV of 0.972, and PPV of 0.881. Although RF showed slightly higher performance in several threshold-dependent classification metrics, XGB demonstrated the best overall discrimination and calibration performance. Therefore, XGB was selected as the final model for subsequent interpretation and clinical risk-stratification analysis.

The identification of CD4 + T cells and CD3 + total T cells as independent factors in the multivariable logistic regression analysis, together with their high SHAP ranking in the XGB model, suggests that alterations in host cellular immunity are closely associated with DI-TB. CD4 + T cells are essential for Th1-mediated anti-tuberculosis immunity, IFN-*γ*-dependent macrophage activation, and granuloma maintenance. Therefore, reduced or functionally impaired CD4 + T cells may weaken local immune containment of *Mycobacterium tuberculosis* and facilitate hematogenous or lymphatic dissemination. Previous studies have reported profound peripheral T-lymphocyte depletion and activation in disseminated tuberculosis (15). This immune impairment may be related to downregulated NF-κB and TNF signaling, which can compromise T-cell activation and macrophage-mediated granulomatous responses (16). Enhanced regulatory T-cell activity may further suppress protective Th1 responses and promote bacterial immune escape (17). In addition, tuberculosis infection may induce lymphocyte apoptosis, contributing to peripheral T-cell depletion and impaired immune surveillance (18). CD3 + total T cells reflect the overall circulating T-cell pool; therefore, their abnormality may indicate broader adaptive immune dysfunction and insufficient antigen-specific immune control in DI-TB. In addition to T-cell-related indicators, CD19 + B-cell-related variables were also identified as important factors associated with DI-TB. We found decreased CD19 + B-cell counts in DI-TB, potentially mediated by multiple mechanisms including FasL–Fas apoptosis (19), B-regulatory (Breg) cell exhaustion (20), altered T-cell/B-cell interaction suppressing Th17 (21), and chemokine-driven redistribution to ectopic lymphoid structures in the lungs (20), suggesting involvement in granuloma formation and neutrophil regulation (22).

From a clinical translation perspective, the XGB model should be positioned as an auxiliary risk-stratification tool rather than a stand-alone diagnostic test. In routine practice, hospitalized TB patients may present with pulmonary symptoms or nonspecific systemic manifestations, while extrapulmonary lesions may remain clinically occult. Indiscriminate use of PET-CT or extensive multi-organ investigations in all patients may increase medical cost and resource burden, whereas simple observation may delay the diagnosis of DI-TB in high-risk patients. Therefore, the proposed model may serve as a pre-test risk assessment tool before PET-CT or confirmatory etiological testing (23, 24). A potential workflow is as follows: routine laboratory indicators and lymphocyte subset parameters are collected at admission from HIV-negative hospitalized TB patients with suspected or confirmed PTB, and then entered into the XGB model to generate an individualized predicted probability of DI-TB. Patients with a low predicted probability may continue standard diagnostic evaluation and close follow-up, whereas patients with a high predicted probability should be considered for intensified evaluation, including PET-CT when available, organ-specific imaging, mycobacterial culture, GeneXpert MTB/RIF, histopathological examination, or sampling from suspected extrapulmonary sites according to clinical presentation (24, 25). This approach may help prioritize patients who are more likely to benefit from further multi-organ assessment while reducing unnecessary examinations in low-risk patients. The model is not intended to provide etiological confirmation or anatomical localization of lesions. Compared with PET-CT, mycobacterial blood culture, and invasive tissue-based examinations, its potential advantage lies in providing an early, non-invasive, and low-burden risk estimate using routinely available laboratory and immune-cell indicators. Therefore, it should be viewed as a triage tool that supports pre-test risk stratification and guides the selection of subsequent diagnostic tests, rather than replacing established diagnostic modalities.

Because the XGB model is intended as an auxiliary triage tool rather than a stand-alone diagnostic test, the choice of threshold should depend on the clinical scenario. In settings where the main goal is early detection and reduction of missed DI-TB cases, a lower threshold may be preferred to increase sensitivity and NPV, although this may increase false-positive classifications and lead to additional imaging or microbiological examinations. In contrast, a higher threshold may improve specificity and PPV, thereby reducing unnecessary examinations, but may miss some patients with early or occult disseminated disease. Based on the DCA results, the model may provide more clinical benefit than the “treat-all” or “treat-none” strategies across a broad intermediate threshold range. Therefore, we do not propose a universal cutoff in this retrospective single-center study. Instead, threshold selection should be prospectively determined according to local DI-TB prevalence, diagnostic resources, PET-CT availability, clinical tolerance for false negatives, and the consequences of additional examinations.

This study has several limitations. First, this was a single-center retrospective study conducted at Nanjing Second Hospital, and only internal validation based on stratified data splitting was performed. Although the training and internal test sets showed consistent model performance, the absence of an external validation cohort remains a major limitation. The model may have captured center-specific characteristics, including local referral patterns, laboratory testing procedures, patient composition, and diagnostic practices. As a result, its performance and clinical applicability in other institutions, regions, or healthcare systems remain uncertain. Therefore, the model should currently be considered an exploratory and development-stage auxiliary diagnostic tool rather than a validated model ready for broad clinical implementation. Potential selection bias related to lymphocyte subset testing should also be noted, as this test was not routinely performed for all admitted TB patients and patients without these measurements were excluded. This may limit the representativeness of the included cohort and the generalizability of the model. In addition, the near-perfect performance of XGB in the training set suggests a potential risk of overfitting, although its performance remained favorable in the internal test set. Future studies should use standardized lymphocyte subset testing, nested cross-validation, and independent external validation to reduce bias and further assess model robustness. Future work will prioritize prospective multicenter validation by enrolling TB patients from hospitals with different geographic regions, laboratory platforms, and clinical practice patterns. The model will be externally assessed using discrimination, calibration, decision-curve analysis, and threshold-based clinical utility metrics. If necessary, model recalibration or updating will be performed before clinical deployment.

## 5 Conclusion

In summary, we developed and internally evaluated a machine learning-based auxiliary risk-stratification model for identifying dissemination risk among HIV-negative hospitalized TB patients. The XGB model showed promising internal performance, and SHAP analysis provided interpretable evidence regarding immune-related predictors. However, given the single-center retrospective design and the absence of external validation, this model should currently be regarded as an exploratory auxiliary tool. Prospective multicenter validation and clinical implementation studies are required before routine clinical application.

## Data Availability

The raw data supporting the conclusions of this article will be made available by the authors, without undue reservation.
