# Supplementary material for: Development of an early auxiliary diagnostic model for disseminated tuberculosis in HIV-negative tuberculosis patients
Source: Front Public Health. 2026 Aug 5;14:1779279. doi: 10.3389/fpubh.2026.1779279 (PMC13491642; doi:10.3389/fpubh.2026.1779279)
Supplement: Supplementary file 1 [file Supplementary_file_1.DOCX]

**Supplemental Materials**

**Table S1. Multivariate logistic regression analysis of factors independently associated with DI-TB**

| **Variable** | **Adjusted OR** | **95% CI** | **P value** |
| --- | --- | --- | --- |
| Male sex | 0.37 | 0.17–0.82 | 0.014 |
| Smoking | 4.70 | 1.20–18.45 | 0.027 |
| Drinking | 0.17 | 0.03–0.93 | 0.041 |
| Absolute lymphocyte count | 7.76 | 1.62–37.08 | 0.010 |
| Monocyte percentage | 1.19 | 1.03–1.38 | 0.020 |
| Absolute neutrophil count | 8.21 | 1.84–36.65 | 0.006 |
| White blood cell count | 0.16 | 0.04–0.64 | 0.009 |
| IL-2 | 1.05 | 1.02–1.08 | 0.002 |
| IFN-γ | 0.99 | 0.98–1.00 | 0.018 |
| CD3+ total T cells | 0.98 | 0.97–1.00 | 0.004 |
| CD8+/CD45+ | 0.84 | 0.75–0.95 | 0.005 |
| CD8+ T cells | 1.02 | 1.01–1.03 | 0.003 |
| CD4+ T cells | 0.99 | 0.98–1.00 | 0.037 |
| CD19+ B cell percentage | 0.83 | 0.72–0.96 | 0.013 |

Note: OR, odds ratio; CI, confidence interval; DI-TB, disseminated tuberculosis; IL, interleukin; IFN-γ, interferon-gamma. Only variables with P < 0.05 in the multivariate logistic regression model are shown.

**Figure S1. Feature selection based on LASSO regression.**

**(A) Cross-validation curve for selecting the optimal penalty parameter λ. The vertical axis represents binomial deviance, and the horizontal axis represents log(λ). (B) LASSO coefficient profile of candidate predictors across different log(λ) values.**

**Figure S2. Calibration curves of the machine learning models.**

(A) **Training set. (B) Internal test set.**

**Figure S3. Decision curve analysis of the machine learning models.**

**“Treat all” represents the net benefit assuming that all patients receive further diagnostic intervention, whereas “treat none” represents the net benefit assuming that no patients receive further diagnostic intervention.**

**Table S2. Performance comparison of the five machine learning algorithms.**

| **Dataset** | **Model** | **Accuracy** | **Sensitivity** | **Specificity** | **PPV** | **NPV** | **F1-score** | **MCC** |
| --- | --- | --- | --- | --- | --- | --- | --- | --- |
| Training set | LR | 0.939 | 0.921 | 0.945 | 0.841 | 0.974 | 0.879 | 0.839 |
| Internal test set | LR | 0.946 | 0.926 | 0.953 | 0.862 | 0.976 | 0.893 | 0.858 |
| Training set | KNN | 0.936 | 0.931 | 0.938 | 0.826 | 0.977 | 0.876 | 0.836 |
| Internal test set | KNN | 0.920 | 0.914 | 0.922 | 0.787 | 0.971 | 0.846 | 0.796 |
| Training set | SVM | 0.952 | 0.942 | 0.955 | 0.868 | 0.981 | 0.904 | 0.873 |
| Internal test set | SVM | 0.943 | 0.901 | 0.957 | 0.869 | 0.968 | 0.885 | 0.848 |
| Training set | RF | 1.000 | 1.000 | 1.000 | 1.000 | 1.000 | 1.000 | 1.000 |
| Internal test set | RF | 0.961 | 0.938 | 0.969 | 0.905 | 0.980 | 0.921 | 0.896 |
| Training set | XGB | 0.999 | 1.000 | 0.998 | 0.995 | 1.000 | 0.997 | 0.997 |
| Internal test set | XGB | 0.949 | 0.914 | 0.961 | 0.881 | 0.972 | 0.897 | 0.864 |

Note: MCC, Matthews correlation coefficient; NPV, negative predictive value; PPV, positive predictive value. Sensitivity is equivalent to recall; PPV is equivalent to precision.

**Table S3. Confusion matrix of the final XGB model in the internal test set**

| **Actual diagnosis** | **Predicted PTB** | **Predicted DI-TB** |
| --- | --- | --- |
| PTB | 245 | 10 |
| DI-TB | 7 | 74 |

Note: XGB, extreme gradient boosting; PTB, pulmonary tuberculosis; DI-TB, disseminated tuberculosis.

**Table S4. Sensitivity comparison of the XGB model with and without SMOTE in the internal test set**

| **Imbalance-handling strategy** | **AUC** | **Accuracy** | **Sensitivity** | **Specificity** | **PPV** | **NPV** | **F1-score** | **MCC** | **Brier score** |
| --- | --- | --- | --- | --- | --- | --- | --- | --- | --- |
| No SMOTE | 0.975 | 0.961 | 0.889 | 0.984 | 0.947 | 0.965 | 0.917 | 0.893 | 0.034 |
| SMOTE k = 5 | 0.983 | 0.949 | 0.914 | 0.961 | 0.881 | 0.972 | 0.897 | 0.864 | 0.025 |

Note: SMOTE was applied only to the training set after stratified data splitting, while the internal test set retained its original class distribution. XGB, extreme gradient boosting; SMOTE, synthetic minority oversampling technique; PPV, positive predictive value; NPV, negative predictive value; MCC, Matthews correlation coefficient.

**Table S6. Simplified TRIPOD+AI checklist for reporting of the prediction model study**

| **Domain** | **Location in manuscript** | **Brief description** |
| --- | --- | --- |
| Study objective and design | Title; Abstract; Introduction; Methods 2.1 | The study is described as a single-center retrospective ML-based prediction model study for auxiliary DI-TB risk stratification. |
| Participants and data source | Methods 2.1; Figure 1 | Eligibility criteria, exclusion criteria, data source, and patient screening process are reported. |
| Outcome definition | Methods 2.1 | PTB and DI-TB definitions are provided; DI-TB is defined as TB involving two or more non-contiguous organs or anatomical sites. |
| Candidate predictors | Methods 2.2 and 2.4 | Candidate predictors included demographic, clinical, laboratory, cytokine, and lymphocyte subset indicators. |
| Missing data and selection | Methods 2.1 and 2.4; Discussion | Patients without lymphocyte subset testing were excluded; complete-case analysis was used for remaining modeling variables; possible selection bias is discussed. |
| Data splitting and leakage control | Methods 2.4 | The dataset was divided into training and internal test sets before model development; the internal test set was used only for final evaluation. |
| Class imbalance | Methods 2.4; Results 3.5; Table S4 | SMOTE with k = 5 was applied only to the training set, and sensitivity analysis with and without SMOTE was performed. |
| Feature selection | Methods 2.4; Results 3.2-3.3; Figure S1 | Backward stepwise regression and LASSO were used; final predictors were selected using an intersection-based strategy. |
| Model development | Methods 2.3-2.4; Results 3.4 | Five ML models were developed and tuned using grid search with five-fold cross-validation. |
| Model performance | Results 3.4; Table S2; Figure 2; Figure S2; Table S3 | Discrimination, calibration, classification metrics, Brier score, and confusion matrix were reported. |
| Clinical utility and threshold use | Results 3.4; Figure S3; Discussion | DCA was performed; the model was positioned as an auxiliary triage tool, and threshold use was discussed clinically. |
| Interpretability | Results 3.6; Figure 3 | SHAP analysis was used to interpret feature importance and feature-specific contributions to XGB predictions. |
| Subgroup performance | Results 3.7; Table 2 | Internal test set subgroup performance was assessed by age, sex, and diabetes status. |
| Risk of bias and limitations | Discussion; Conclusion | Single-center design, lack of external validation, selection bias, possible overfitting, and implementation limitations are discussed. |
| Data availability | Availability of data materials | Data availability is stated according to journal requirements. |

Note: TRIPOD+AI, Transparent Reporting of a multivariable prediction model for Individual Prognosis Or Diagnosis-Artificial Intelligence; DI-TB, disseminated tuberculosis; PTB, pulmonary tuberculosis; ML, machine learning; SMOTE, synthetic minority oversampling technique; XGB, extreme gradient boosting; SHAP, Shapley Additive Explanations; AUC, area under the receiver operating characteristic curve; DCA, decision curve analysis.
